# Progression of Behavioral and CNS Deficits in a Viable Murine Model of Chronic Neuronopathic Gaucher Disease

**DOI:** 10.1371/journal.pone.0162367

**Published:** 2016-09-06

**Authors:** Mei Dai, Benjamin Liou, Brittany Swope, Xiaohong Wang, Wujuan Zhang, Venette Inskeep, Gregory A. Grabowski, Ying Sun, Dao Pan

**Affiliations:** 1 Experimental Hematology and Cancer Biology, Cincinnati Children’s Hospital Medical Center, Cincinnati, Ohio, United States of America; 2 Division of Human Genetics, Cincinnati Children’s Hospital Medical Center, Cincinnati, Ohio, United States of America; 3 Department of Pediatrics, University of Cincinnati School of Medicine, Cincinnati, Ohio, United States of America; 4 Division of Pathology, Cincinnati Children’s Hospital Medical Center, Cincinnati, Ohio, United States of America; National Institutes of Health, UNITED STATES

## Abstract

To study the neuronal deficits in neuronopathic Gaucher Disease (nGD), the chronological behavioral profiles and the age of onset of brain abnormalities were characterized in a chronic nGD mouse model (9V/null). Progressive accumulation of glucosylceramide (GC) and glucosylsphingosine (GS) in the brain of 9V/null mice were observed at as early as 6 and 3 months of age for GC and GS, respectively. Abnormal accumulation of α-synuclein was present in the 9V/null brain as detected by immunofluorescence and Western blot analysis. In a repeated open-field test, the 9V/null mice (9 months and older) displayed significantly less environmental habituation and spent more time exploring the open-field than age-matched WT group, indicating the onset of short-term spatial memory deficits. In the marble burying test, the 9V/null group had a shorter latency to initiate burying activity at 3 months of age, whereas the latency increased significantly at ≥12 months of age; 9V/null females buried significantly more marbles to completion than the WT group, suggesting an abnormal response to the instinctive behavior and an abnormal activity in non-associative anxiety-like behavior. In the conditional fear test, only the 9V/null males exhibited a significant decrease in response to contextual fear, but both genders showed less response to auditory-cued fear compared to age- and gender-matched WT at 12 months of age. These results indicate hippocampus-related emotional memory defects. Abnormal gait emerged in 9V/null mice with wider front-paw and hind-paw widths, as well as longer stride in a gender-dependent manner with different ages of onset. Significantly higher liver- and spleen-to-body weight ratios were detected in 9V/null mice with different ages of onsets. These data provide temporal evaluation of neurobehavioral dysfunctions and brain pathology in 9V/null mice that can be used for experimental designs to evaluate novel therapies for nGD.

## Introduction

Gaucher disease (GD) is an autosomal recessive lysosomal storage disorder with a broad spectrum of severities. In GD, mutations of *GBA1* lead to defective function of acid β-glucosidase (GCase) and subsequent accumulation of its substrates, glucosylceramide (GC) and glucosylsphingosine (GS) [1]. Accumulation of these substrates affects normal cell function and promotes disease progression in the viscera and central nervous systems (CNS) [1–4]. Over 350 *GBA1* mutations have been identified [5, 6]. Most of the mutations can be found in patients with varying degrees of visceral and/or CNS manifestations that are classified as type 1, type 2 or type 3 variants [1, 7, 8]. Patients with GD type 1 do not exhibit any early-onset progressive CNS abnormalities, but develop hepatomegaly, splenomegaly, bone pain and fractures, growth retardation, anemia and thrombocytopenia with highly variable penetrance and presentation [1]. GD type 2 is an acute neuronopathic disease with onset in the first few months of life and progression to death between 3 and 24 months. In addition to visceral involvement, GD type 2 patients have progressive CNS manifestations that include bulbar signs, ataxia, and seizures [1, 8]. GD type 3 patients present various signs of neuronopathic and visceral involvement with chronic progression, and may survive into the 2^nd^ to the 5^th^ decades of life [1, 4]. Currently, two therapeutic approaches are approved for the visceral manifestations of GD, i.e. enzyme replacement therapy (ERT) and substrate reduction therapy (SRT) [9, 10]. However, there have been no effective treatment options for the neurological sequelae of GD patients and innovative therapies are still needed.

Experimental and epidemiological evidence have strongly implicated an excess risk of Parkinson’s disease (PD) and Lewy body disease in GD type 1 patients, as well as heterozygote carriers of *GBA1* mutations [11–15]. Indeed, *GBA1* GD-causative mutations are broadly recognized as the most common genetic risk factor for the development of Parkinsonism and Lewy Body disease that not only increases susceptibility to PD, but also drives the disease progression with an earlier onset or increased severity [12, 16–21]. The risk of developing PD in otherwise healthy carriers of *GBA1* mutations is estimated to be 13.7% at 60 and 29.7% at 80 years of age, significantly higher than in the general population [22]. The mechanism of the connection between GD and PD has not been fully elucidated, although lysosomal and/or mitochondrial dysfunctions with subsequently impaired autophagy have been indicated [21, 23–25]. The clinical and pathogenic heterogeneity of GD is a continuum of disease progression with a difference in the presence or absence of neurologic involvement that may present as an acute or chronic course [26–28]. Identification of the phenotypic expression with diverse manifestations in nGD would be valuable to facilitate the understanding of some conditions that are widespread in all populations. However, little is known about the longitudinal course of biochemical and neurological defects in chronic nGD.

Several transgenic mouse models with *Gba1* mutations have been generated and display defective GCase activity, including those with homozygosity for L444P, R463C, V394L, D409V, or D409H and D409V/null (9V/null) [29–31]. These mutant mice have a nearly normal lifespan (~2 years) with moderate visceral abnormalities and substrate accumulation. Interestingly, an abnormal neurological phenotype occurs in the mouse model homozygous for the D409V mutation which displays elevated α-synuclein (αSyn) pathology concomitant with memory deficit by one year of age [32], indicating this mouse is representative of some defects seen in chronic nGD patients. However, the progressive deterioration of CNS function with age has not been addressed. Comprehensive evaluation of phenotypic expression in a chronic nGD mouse model can provide valuable information because of the progressive nature of the disease, gender differences, and complications arising from hematological and visceral symptoms on CNS pathology and behavioral appearance. In the present report, the chronological profiles of biochemical and behavioral changes as well as the age of onset of CNS abnormalities are identified in the 9V/null mice, a chronic nGD mouse model. The progressive CNS pathogenesis and behavioral abnormalities characterized in 9V/null mice would provide important guideline for future evaluation of novel therapies in chronic nGD.

## Materials and Methods

### Materials

The following were from commercial sources: Anti-β-actin monoclonal antibody (Sigma, St. Louis, MO). SuperSignal™ West Dura (ThermoFisher Scientific, Rockford, IL). Anti-mouse αSyn monoclonal antibody (Novus Biologicals, Littleton, CO). Anti-mouse αSyn monoclonal antibody (Abcam, Cambridge, MA). Goat anti-mouse Alex 595 (Invitrogen, Grand Island, NY). Goat-anti-mouse IgG-HRP (Cell signaling, Danvers, MA). Tyramide Signal Amplification (TSA) kit (Perkin Elmer, Waltham, MA). C16-, C18- and C24:1-GCs and C8-GC (Avanti Polar Lipids, Inc., Alabaster, AL).

### Mouse model and tissues collection

D409V/null mice were generated by crossing D409V/D409V (referring to amino acid location in mature protein sequence) with *Gba1* null/WT mice [29]. The 9V/null mice are hetero-allelic for *Gba1* alleles encoding a Valine for Aspartate (9V) and null mutations. The 9V/null and WT mice used in these studies are a mixture of C57/BL6, 129SvEvBrd and FVB strain backgrounds and genotyped as described [29]. The mice were maintained in micro-isolators in accordance with institutional guidelines under IACUC approval at Cincinnati Children’s Hospital Research Foundation. The protocol includes early/humane endpoints requiring that mice be euthanized if they are unable to drink, eat or move, or have lost 20% of body weight. No animals died prior to the experimental endpoint. Mice were housed in 12 light/12 dark cycles and fed with pelleted chow.

The mice were anesthetized with sodium pentobarbital and euthanized by transcardiac perfusion with cold phosphate-buffered saline (PBS) before tissue dissection. Portions of dissected tissues were fixed in 4% paraformaldehyde (PFA) in PBS for histology analysis. The remaining portions were snap-frozen for immunobloting, enzyme assays and lipid analyses. Body weights were taken before perfusion, and organ weights were recorded at dissection.

### Immunofluorescence

Frozen sagittal brain sections from WT and 9V/null mice were fixed (4% PFA, 30 min) and permeabilized (0.3% Triton X-100, 20 min). The sections were treated with proteinase K (10 μg/mL) for 7 min in TNB buffer (TSA kit, PerkinElmer NEL705A001) to remove soluble αSyn from tissue sections. The sections were incubated with anti-mouse αSyn monoclonal antibody (Abcam 3H9, 1:200 dilutions) overnight at 4°C. Fluorescent signals were detected by goat anti-mouse Alex 595 (Invitrogen). Fluorescence signals were visualized and captured by Zeiss Axiovert 200 M microscopy equipped with an Apotome.

### Immunoblot

Brain tissues (1:10, w/v) were homogenized in lysis buffer (10 mM Tris-Cl, 1 mM EDTA, 0.5 mM EGTA, 140 mM NaCl, pH 8.0) containing Protease Inhibition Cocktail III. Soluble and insoluble αSyn proteins were subsequently lysed with two different detergents. The soluble part was first extracted with 1% Triton X-100 in the lysis buffer. The insoluble part in the remaining pellet was further extracted with lysis buffer containing 1% Triton X-100 /2% SDS. Protein concentration was determined by BCA Protein Assay kit (Thermo Fisher Scientific). Pre-casted 4–12% gradient gel (Invitrogen) was used for protein separation. The proteins on the gels were transferred onto 0.45μm PVDF membranes. The membrane was blocked in 5% milk in PBS for 20 min at room temperature. Anti-mouse αSyn monoclonal antibody (1:500 in 3% milk/PBS, Novus) was incubated overnight at 4°C followed by goat-anti-mouse IgG-HRP (1:1500 in 1% milk/PBS, Cell signaling) incubation for 1 hr at room temperature. An ECL detection system (SuperSignal™ West Dura, Thermo Fisher Scientific) was applied for signal detection. Anti β-actin monoclonal antibody (1:5000, Sigma) was used as a loading control.

### Lipid analyses

Mouse brain tissues were homogenized in methanol/chloroform/water (3.6 mL, 2:1:0.6, v/v/v). Glycosphingolipids were extracted and subjected to alkaline methanolysis and followed by elution from Sephadex G-25 fine columns to remove non-lipid contaminants [33, 34]. The extracted samples were re-dissolved in methanol containing an internal standard. GC and GS analyses were carried out with ESI-LC-MS/MS using a Waters Quattro Micro API triple-quadrupole mass spectrometer (Milford, MA) interfaced with an Acquity UPLC system [33, 34].

### Repeated open-field test

The repeated open-field test was performed with mice at different ages that were naïve to the test as described [35]. The open-field apparatus (60 x 60 cm) consisted of a white Plexiglas box with 25 squares (12 x 12 cm) painted on the floor (16 outer and 9 inner). Briefly, each mouse was placed in one of the four corners of the apparatus and allowed to explore for 5 min. Activity was monitored and quantified for ambulation (number of squares crossed) and time spent grooming by two observers in blinded experiments. Each mouse was tested for three repeated trials with 30-minute inter-trial intervals.

### Marble burying test

The marble burying test for anxiety-like behaviors was performed at multiple ages [36]. Mice were individually placed into cages (28×17×12 cm) filled with wood chip bedding to a depth of 3 cm for a 30-min testing period. Prior to each testing round, the experimenter evenly spaced 15 identical marbles across the bedding surface using a template. Animals were given 30 min of exposure to the marbles, and the dependent measures were latency to begin the burying activity as well as the number of marbles visible at the end of the test. A fewer number of visible marbles (i.e., a greater number of buried marbles) was considered an index of greater anxiety/compulsive-like behavior.

### Auditory fear conditioning test

Cued and contextual fears were assessed as described previously with modification [37, 38] in Animal Behavioral Core at Cincinnati Children’s Medical Center. During habituation process on Day 1, mice were placed in the test chambers (SDI, San Diego, CA) for 6 min prior to tone onset while locomotor activity was measured, and followed by 3 tones/shock pairings (82 dB, 2 kHz, 30 s duration, co-terminating the tone interval with 1 s of 0.5 mA footshock delivered through the grid floor). Tone-shock pairings were separated by 135 s intervals. On day 2, animals were returned to the same chamber for 6 min without any tone or shock to assess contextual freezing. On day 3, mice were placed in a dark acrylic triangular box that fits within the test chambers. The test session was 6 min, with no tone during the first 3 min and the 82 dB tone presented continuously during the last 3 min. Data for contextual fear were analyzed as the percentage of time freezing on day 2. Data for cued conditioning were analyzed as the percentage freezing on day 3 after tone presentation compared with the 3 min prior to tone.

### Paw print test for gait analysis

A transparent runway was lined with a sheet of white paper [39]. Mice with front- and hindpaws painted with different colors of water-soluble non-toxic paint were allowed to walk across the runway 2–3 times. The footprints of each walk were analyzed for stride length (left), base lengths, and distance of overlap of the paws. Finally, four parameters were measured from the footprint pattern to describe the locomotion of the mouse: front-paw and hind-paw base width, stride between right front-paw footprints, and overlap (distance between the middle of left high-paw and left front-paw).

### Data analyses

Data were analyzed with Student’s t-test using GraphPad Prism 5 software. ANOVA test was conducted in fixed effect model with the Kenward-Roger method for the conditioned-fear test.

## Results

### Progressive substrate accumulation in 9V/null brain

**Fig 1:**
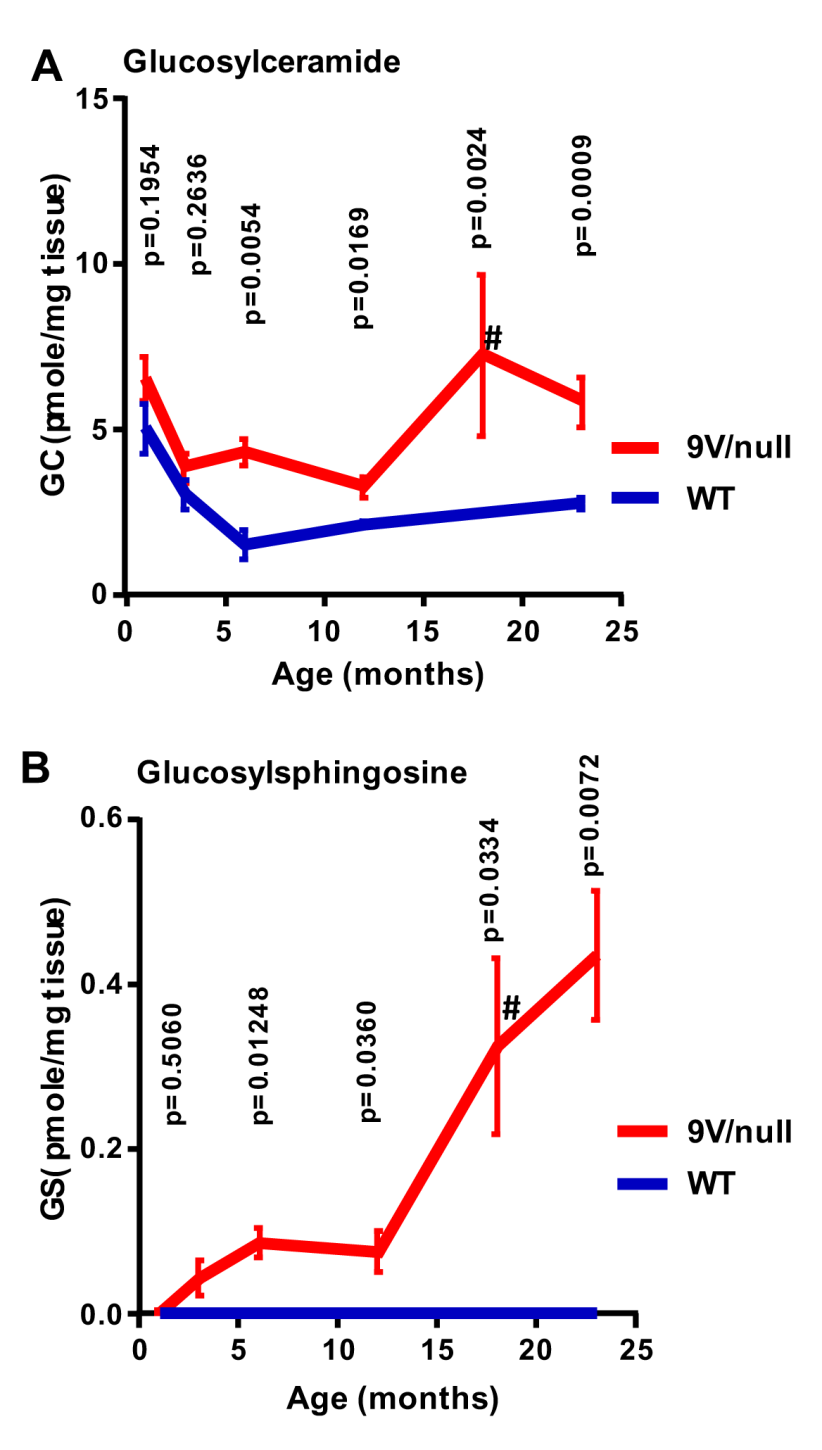
Substrate (GC and GS) levels in 9V/null brain/cortex. A: GC concentrations were significantly increased in 9V/null brain/cortex after 6 months of age compared to age-matched WT mice. B: GS levels were significantly increased in 9V/null brain/cortex with age compared to WT. GS in WT mice brain were at or under detection level. #, data from 18 months-9V/null mice was compared to that from 24 months-WT. All data are expressed as mean ± SEM. P-values were derived from Student’s t-test (n = 4–7 mice/age group except for 12 months WT with 2 mice).

GC and GS in 9V/null and WT brains were analyzed in the brain extracts from well-perfused animals at 6 time points from 1 month to 24 months of age. In WT mice, GC concentrations were highest in the brains of 1-month old mice and steadily decreased to relatively constant levels by 6 months of age (Fig 1). The GC concentrations in 9V/null brain were comparable to those of WT mice at 1 and 3 months and became significantly higher than those of age-matched WT at ≥6 months. Undetectable GS levels were found in the WT brains at all age-points. In comparison, elevated GS concentrations were evident in 9V/null brains at 3 months of age, and continuously increased up to 24 months. The highest GC and GS levels were detected in the brain of 9V/null mice ≥18-months. These results demonstrated that the substrates of GCase accumulated in 9V/null brain during development and were significantly above the WT levels after 3 months of age, indicating progressive metabolic abnormalities in 9V/null brains.

### Repeated open-field habituation

**Fig 2:**
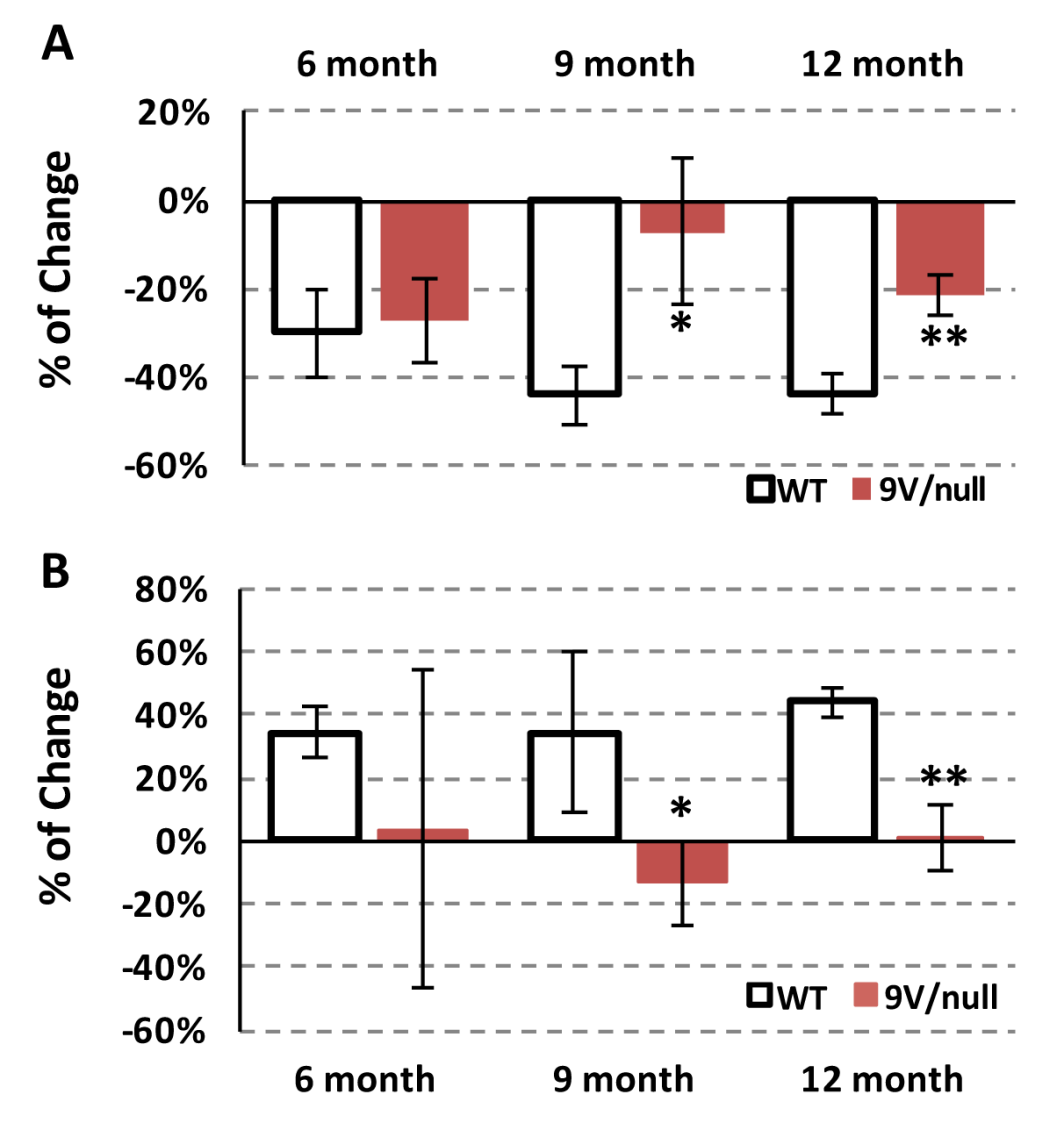
Short-term memory deficits in 9V/null mice evaluated by repeated open-field test. Each mouse was allowed to freely explore the arena for three trials with 5 min each and 30-min intervals. Exploring activities in the third trial were compared to the second trial presented as percentage for change of horizontal activity (A) and time spent grooming (B). 9V/null mice showed significantly less habituation in exploring horizontal activity and reduced grooming time at 9 and 12 months of age. *, p<0.05; **, p<0.01 (n = 9–35 mice per age group) by Student’s t-test.

To evaluate potential memory deficits, repeated open-field tests were conducted with mice at 6, 9 and 12 months of age by exposing them to the same open-field environment 3 times with 30 min intervals (Fig 2). The WT mice showed a 44% reduction in exploratory locomotor activity in the 3^rd^ trial after being acclimated to the apparatus at 9 and 12-months of age; whereas age-matched 9V/null mice showed a significantly less reduction (5–21%) in activity (p < 0.05) (Fig 2A). The reduction in exploratory activity was associated with an increase (37–43%) in time spent grooming in WT control animals at all age points tested (Fig 2B). In comparison, the 9V/null mice exhibited minimal changes in grooming time between trials at all age points, reaching a significant difference from WT mice starting at 9 months of age (p < 0.01) (Fig 2B). These results demonstrated abnormal habituation to the environment by the 9V/null group, suggesting neurological deficits in short-term, non-aversive/non-associative memory with the age of onset at 9 months old.

### Conditioned fear test

**Fig 3:**
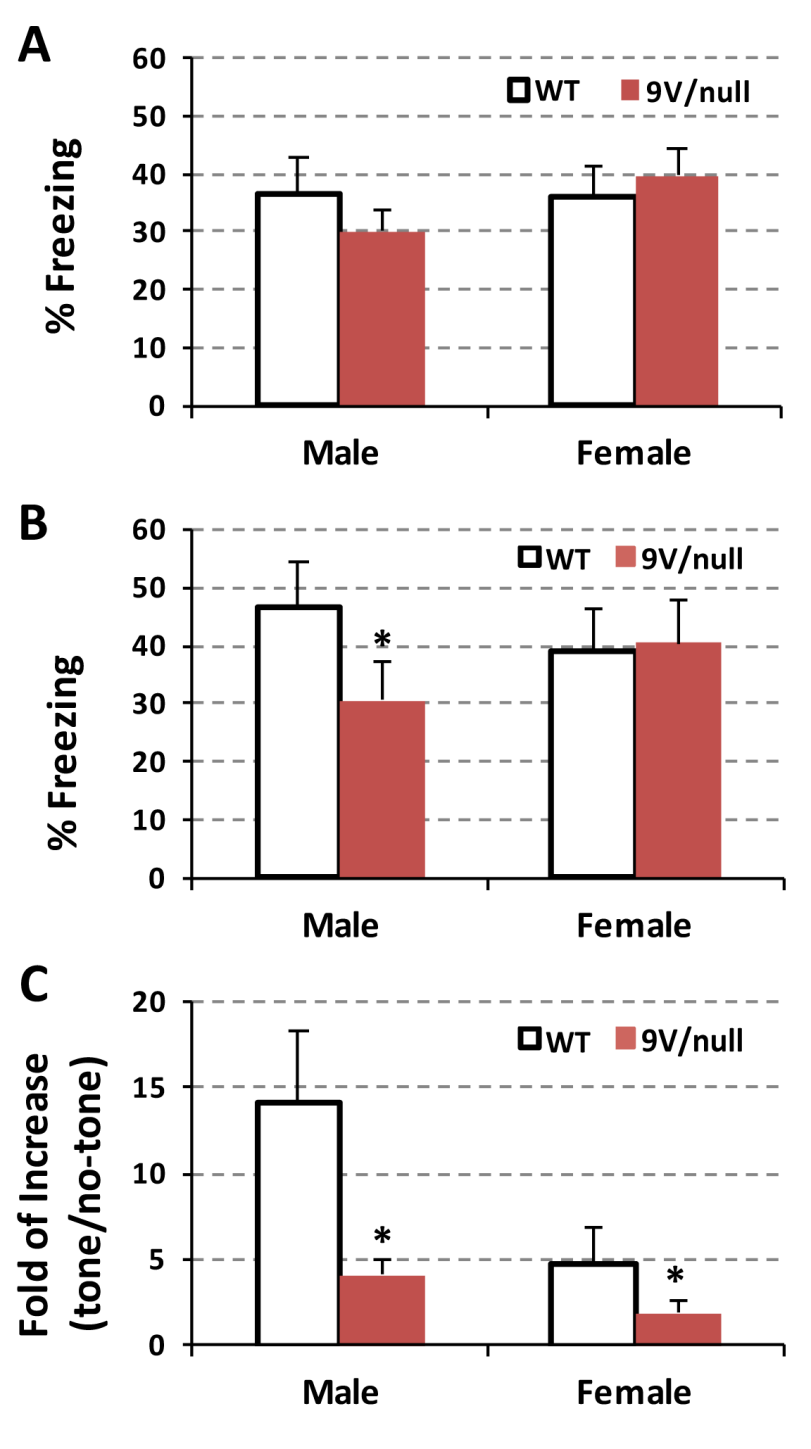
Hippocampus-related memory defects in 9V/null mice evaluated by conditional fear test. A: The mice at 12 months of age were trained to tone-shock pairings on Day 1 in the test chamber for 6 min and percentage of freezing time were quantified. B: On Day 2 (contextual) they were tested for freezing in the same environment without tone and data was presented as percentage of freezing events. C: On Day 3 (cued), a dark acrylic triangular box was placed within the test chamber. After habituation without tone, mice were exposed to the tone. Freezing events were measured and presented as fold of changes with the tone verse without the tone. 9V/null mice exhibited a significant decrease in response to contextual fear in male and auditory-cued fear in both genders compared to age- and gender-matched WT at 12 months of age. *, p<0.05 (n  =  11–12 mice per group) by two-way ANOVA.

To assess emotional memory function, mice were evaluated for auditory fear at 12 months of age (Fig 3). On day 1, mice were trained to tone-shock pairings, showing similar freezing baseline between diseased and normal mice (Fig 3A). On day 2, the male 9V/null mice spent significantly less time freezing than male WT mice (p = 0.037) when exposed to the same environment without the tone (contextual fear) (Fig 3B). Such difference was not observed in female 9V/null mice (p = 0.857). This result suggests that male 9V/null mice remembered to a lesser degree the place where they were shocked compared to 9V/null females or WT of both genders. On day 3, the animals were placed into a new environment and assessed for freezing without any tone, and then with a tone (cued fear) (Fig 3C). WT mice froze instantly and accumulatively longer with the tone than those without the tone (mean of 14-fold for male and 4.8-fold for female), thereby implying that the mice remembered that the tone was associated with a shock. However, the 9V/null mice exhibited significantly less response to tone (mean of 4.1 for male and 1.8 for female). When analyzed using a mixed procedure ANOVA, there was a significant main effect by genotype (F_(1, 19)_ = 6.43, p = 0.020) and by gender (F_(1, 19)_ = 5.08, p = 0.036), and no interaction for the tone-shock pairing presentations (F_(1, 19)_ = 1.94, p = 0.179). The data indicate an abnormal decrease in response to cued fear in 9V/null mice and to contextual fear in the males only, indicating amygdala/hippocampus-related memory defects.

### Marble burying task

**Fig 4:**
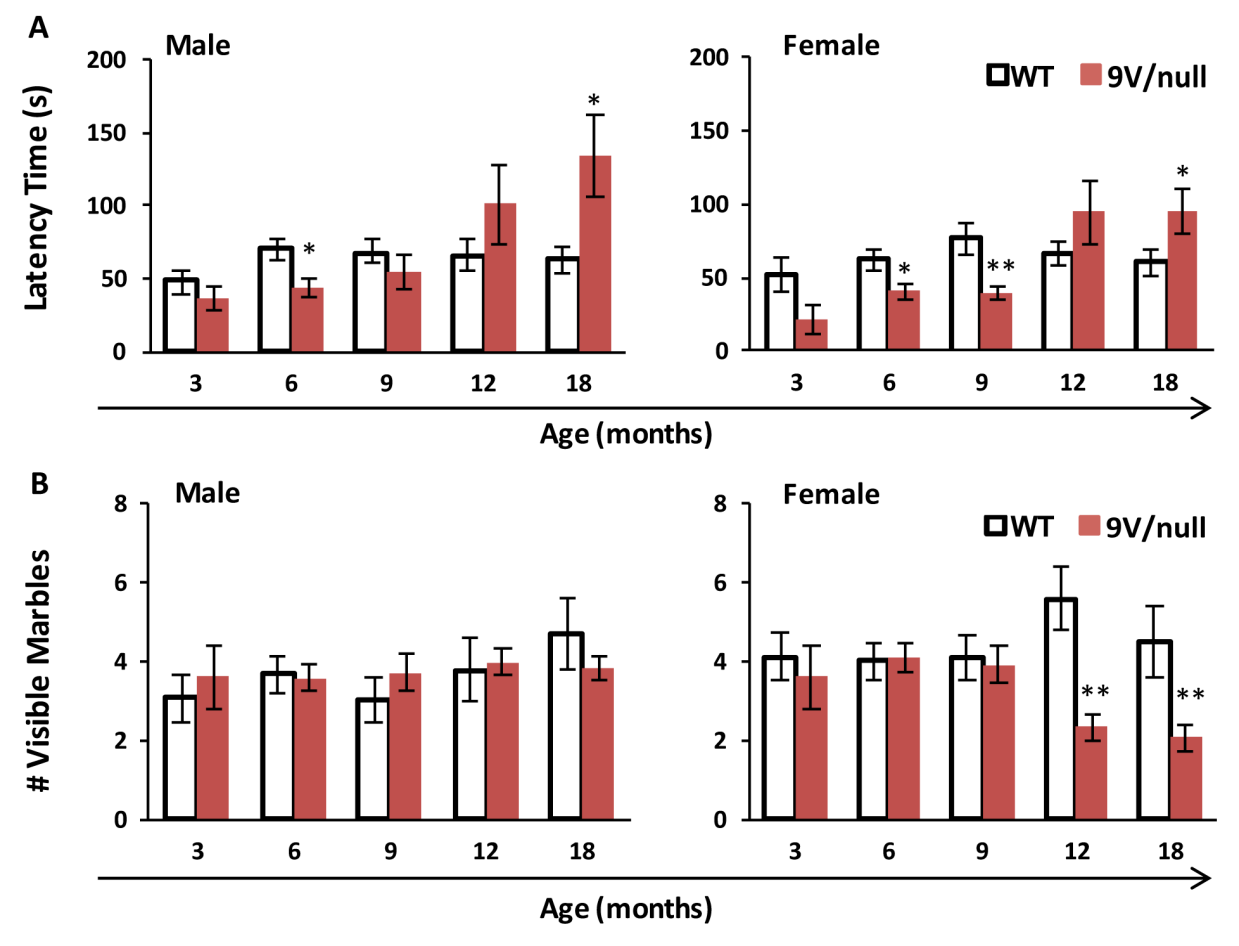
Anxiety-like marble burying behavior in 9V/null mice. Mice were exposed to an arena with 15 blue marbles for 30 min. The test was repeated multiple times from 3 to 18 months of age. A: Latency to initiate burying. B: Number of visible marbles. 9V/null group had a shorter latency to initiate burying activity at 6 months of age, but the latency increased significantly starting at 12 months of age. 9V/null female mice buried significantly more marbles to completion than the WT group. *, p<0.05; **, p <0.01 (n = 8–10 mice for 3 months groups, and n = 12–20 mice for other age groups) by Student’s t-test.

To evaluate the 9V/null mice for anxiety-related and compulsive-like behaviors, the marble burying test was conducted with mice repeatedly at 3, 6, 9, 12 and 18 months of age (Fig 4). The 9V/null group exhibited a shorter latency to initiate burying activity than the WT group at younger ages that reached significance by 6 months of age in both genders; however, their latency increased gradually and became significantly longer than WT littermates at 18 months of age, while similar latency time was observed in WT groups of 6 months and beyond (Fig 4A). In male mice, no differences were observed between genotypes for the number of visible marbles at the end of the 30-min testing period at all ages (Fig 4B). Female 9V/null mice buried significantly more marbles than control females starting at 12 months of age and thereafter (p < 0.01), suggesting increased anxiety. These data imply an abnormal response of 9V/null mice (especially in female), with biphasic phenotypic expression between early and late onsets (latency time), to the instinctive, non-associative, and anxiety-like behavior.

### Gaiting analysis

**Fig 5:**
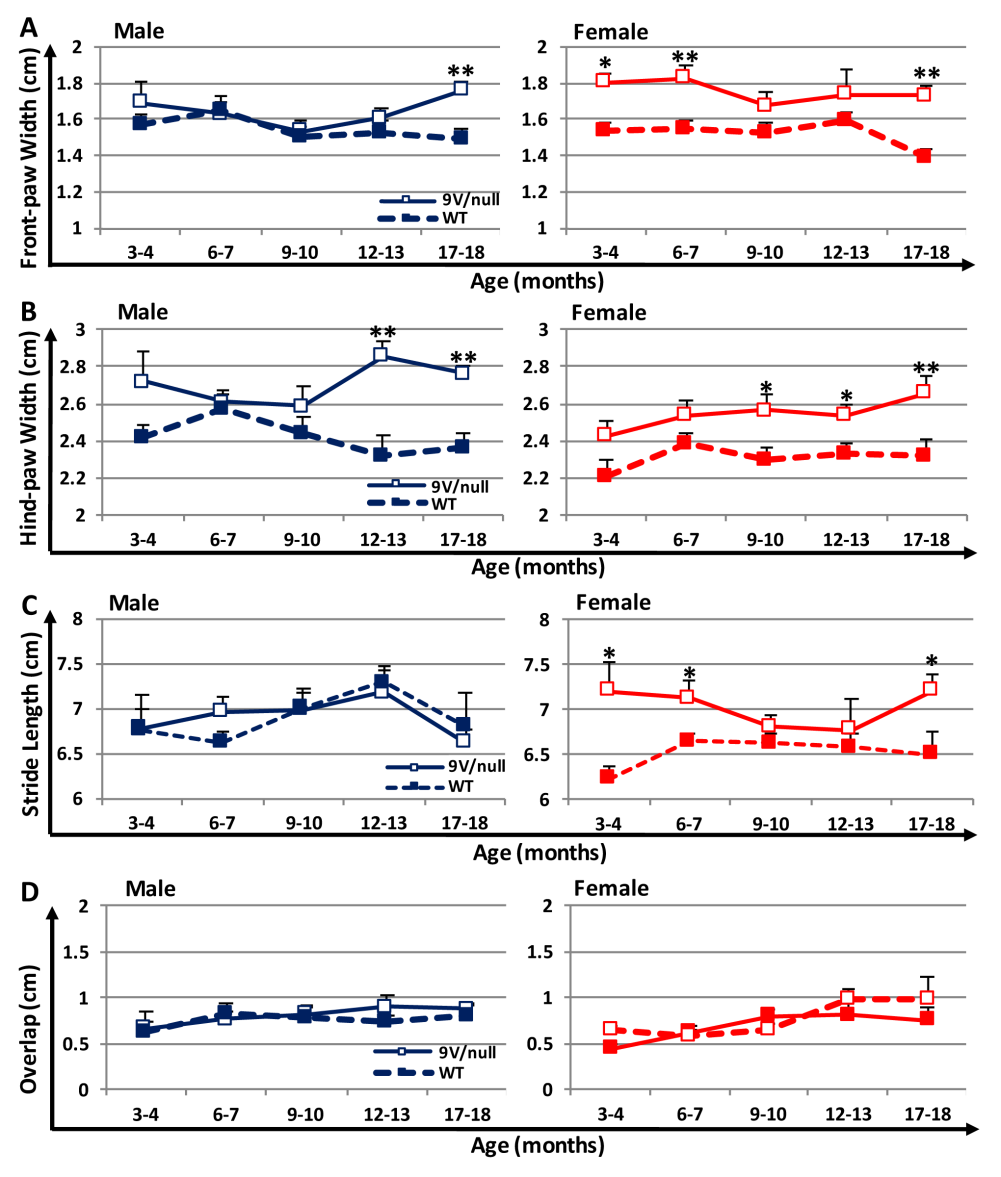
Gait analysis in mice at different ages. Mice painted with different colors on front and hind paws were allowed walking through a clear tunnel at ages indicated. Footprints were measured for front-paw base width (A), hind-paw base width (B), stride length (C), and overlap between the left front and hind paws (D). *, p<0.05; **, p<0.01 (n = 7–25 mice per group) by Student’s t-test.

To evaluate potential movement impairment in 9V/null mice, gait analysis was conducted using gender- and age-matched mice at various ages (Fig 5). Wider front-paw base width between front paws was observed in female 9V/null mice as early as 3 months of age (18% wider than WT, p = 0.021) and thereafter (up to 25% wider, p = 0.0016 at 18 months), and in male mice only at the oldest age tested (18-months, 18% wider than WT, p = 0.0015) (Fig 5A), even though the diseased mice had similar or less body weights than those of normal mice at all ages evaluated (S1 Fig). The hind base width was also wider for the 9V/null mice than for WT in both genders and reached statistical significance at 9 months of age for female (12% wider than WT, p = 0.028) and at 12 months for male mice (23% wider than WT, p = 0.0019) (Fig 5B). Interestingly, female 9V/null mice exhibited a trend of longer stride length than their WT counterparts as early as 3 months of age, reaching significance at 3, 6 and 18 months (16%, 8% and 11% longer than WT, respectively) (Fig 5C). However, stride length was indistinguishable between male 9V/null and WT mice at all ages tested. There were no differences in the overlap of paw placements between genders or genotypes (Fig 5D). These results suggest an abnormal gait, which is more pronounced in females than in males of 9V/null mice, as seen by wider hind-paw and front-paw bases or longer stride length with different ages of onset.

### Synucleinopathy in 9V/null brain

**Fig 6:**
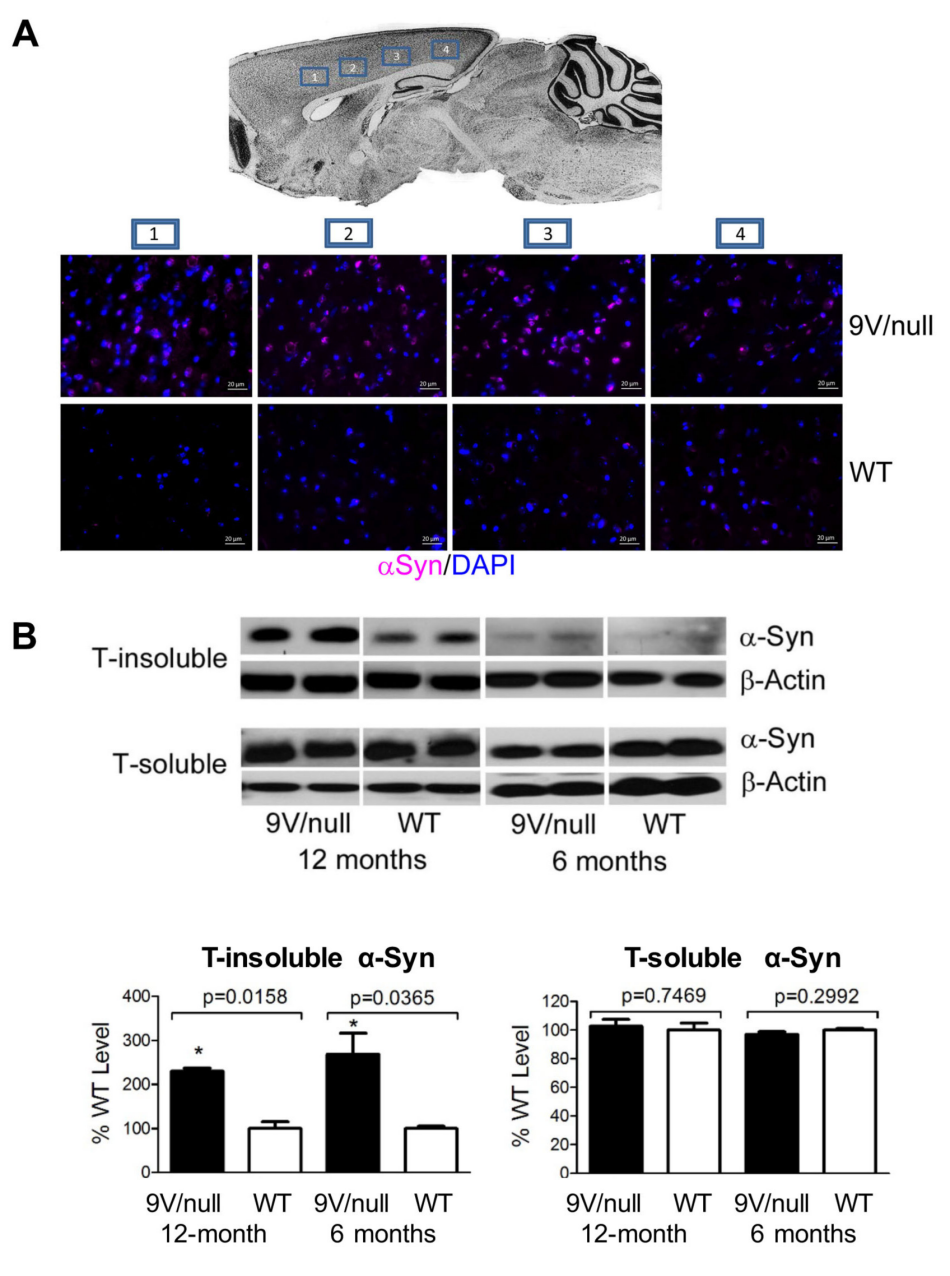
α-Synuclein (αSyn) pathology. A: αSyn aggregates in cortex. Brain map shows four images taken from areas within cortex region including 1) motor, 2) somatosensory, 3) auditory, and 4) visual (upper panel). Representative images are shown with αSyn (violet) signals detected in all 4 brain regions of 9V/null and WT mice at 12 months of age. DAPI (blue) stained nuclei. Scale bars represent 20 μm. B: Immunoblot of αSyn. Upper panel, Triton X-100 insoluble (T-insoluble) αSyn and Triton X-100 soluble (T-soluble) αSyn in the cortex of 9V/null and WT mice at 6 months and 12 months of age, respectively. Lower panel, semi-quantitation of T-insoluble and T-soluble αSyn levels. 9V/null cortex had significantly increased T-insoluble αSyn at 6 and 12 months of age compared to age-matched WT cortex. The levels of T-insoluble αSyn in 6 month-cortex were lower than that in 12 month-cortex. No difference in T-soluble αSyn were found between 9V/null and WT at 6 and 12 months of age. The blots were derived from 3–4 experimental repeats, n = 2–3 mice/group. Intensity of protein bands on the blot were quantified by NIH Image J. The intensity of αSyn was normalized by intensity of β-actin for each sample. The αSyn level in 9V/null was presented as percentage of WT level at each age. P-values are from Student’s t-test.

To evaluate chronological elevation of brain αSyn protein, which is central to PD and GD-linked PD pathology, immunohistostaining and immunoblot analyses were conducted with brain samples of 9V/null and WT mice at 6 and 12 months of age (Fig 6 and S2 Fig). Immunofluorescence staining showed that αSyn aggregates were distributed mainly in cerebral cortical (Fig 6A) and hippocampal (S2A Fig) regions of 9V/null mice and to a lesser extent in other regions: cerebellum and thalamus (S2A Fig). Regions of the cortex with αSyn aggregates include motor, somatosensory, auditory and visual regions (Fig 2A). The αSyn aggregates (size >2 μm) were detected in a portion of neurons or astrocytes, with more positive αSyn signals in 9V/null brains than in WT. These differences reached significance in neurons, but not in astrocytes, at 12 months of age (S2B Fig). The p-Tau signal, a marker for neurodegeneration, was stronger in the cerebral cortex, hippocampus and cerebellum (molecular and granule layer) of 9V/null compared to WT (S2C Fig). Triton X-100 (T)-soluble and -insoluble αSyn proteins were quantified by immunoblot. Significant elevation of T-insoluble αSyn protein was detected in the 9V/null cortex at 6 months and 12 months of age compared to age-matched WT cortex, with an age-dependent increase regardless of genotype (Fig 6B). There was no significant difference in soluble αSyn levels between WT and 9V/null at 6 and 12 months of age (Fig 6B). In addition, no differences in brain cells positive for CD68, a pro-inflammation marker, were observed in the 9V/null brain at 24 months of age as compared with that in the WT brain (S3 Fig). These data demonstrate that synucleinopathy, not inflammation, would play a key role in contributing to neuropathology in the brains of 9V/null mice.

### Organ weight abnormality

**Fig 7:**
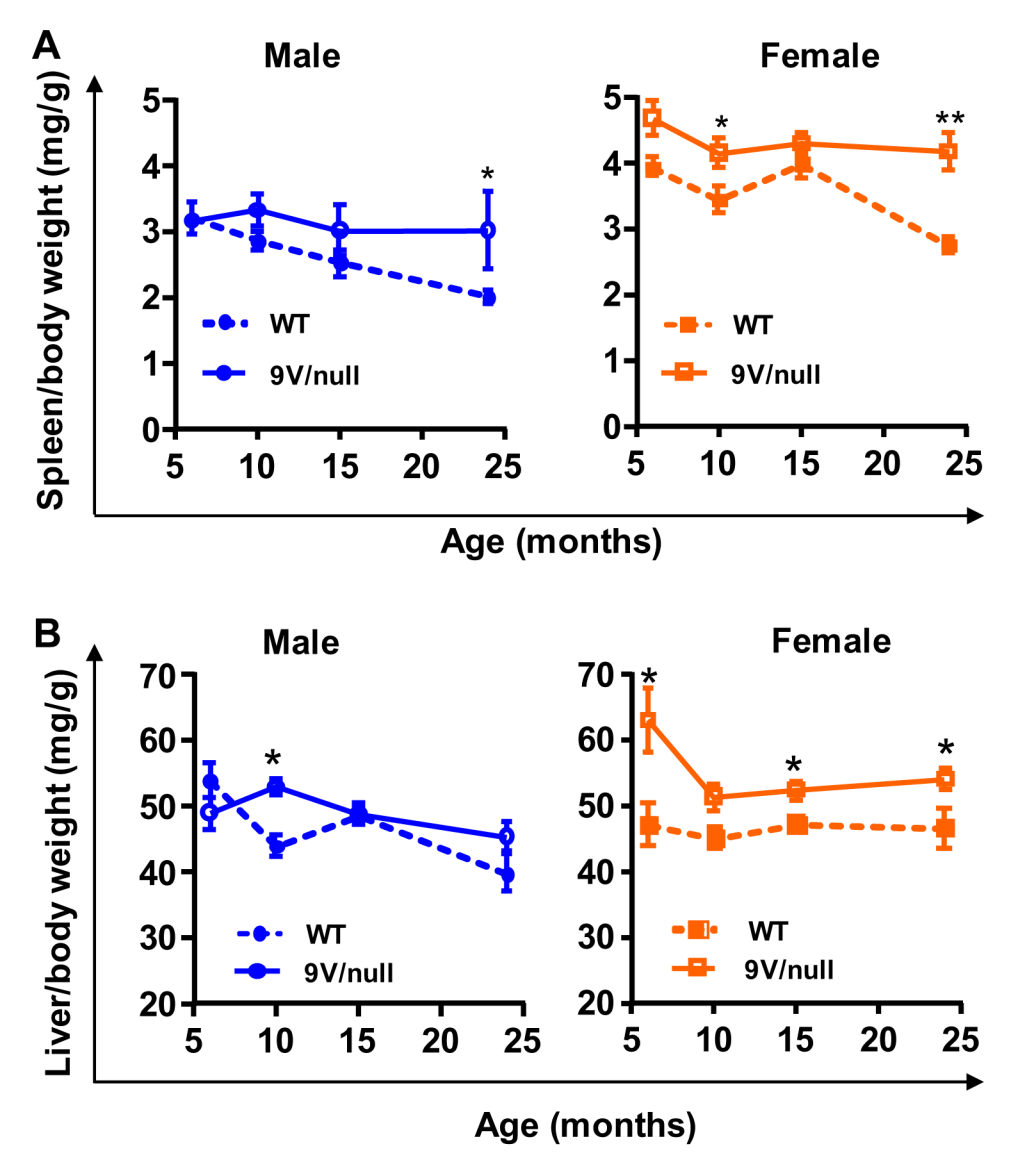
Changes in organ weight ratios. The spleen (A) and liver (B) weight presented as ratio of body weight were significantly increased in female 9V/null mice at the age of 9 months and older compared to age matched WT mice. *, p<0.05; **, p<0.01 (n = 6–16 mice per age group) by Student’s t-test.

Enlarged spleens and livers are often seen in GD patients. To examine if hepatosplenomegaly exists in 9V/null mice model, spleen and liver weights were recorded and analyzed as ratios to body weights in the mice at 6 months through 24 months of age (Fig 7). For the spleen, there were significant gender differences during development within each genotype, with females showing a higher spleen-to-body weight ratio than male mice (p<0.0001 by ANOVA) (Fig 7A). In comparison to WT mice, 9V/null mice had significantly higher spleen-to-body weight ratios in females at as early as 6-months of age (F_(1, 36)_ = 21.33, P<0.0001), and in males at 23 months of age (F _(1, 40)_ = 6.158, p = 0.017). For the liver-to-body weight ratio, there was no gender difference in WT mice, but a significant difference in 9V/null mice (higher in female, p = 0.002) (Fig 7B). Importantly, 9V/null mice exhibited significantly increased liver-to-body weight ratios compared to age-matched WT controls in females (F_(1, 36)_ = 30.76, P<0.0001) starting at 6 months of age, and males (F_(1, 40)_ = 4.518, P = 0.039) only at 24 months of age. These results suggest gender-dependent and age-dependent spleno- and hepatomegaly in 9V/null mice. The clinical manifestations of GD patients often involve bruising, fatigue, anemia and low blood platelet count [40, 41]. Yet, in comparison to age-matched WT mice, the 9V/null mice exhibited comparable levels of red blood cell counts, and similar or higher blood platelet counts at all ages tested (S1 Table).

## Discussion

In this study, the longitudinal and progressive courses of biochemical, histopathological and behavioral CNS abnormalities were evaluated in 9V/null mice with consideration of potential sexual dimorphism. Previous studies on this model have shown storage cells and substrate accumulations in the visceral tissues but a normal life span [29, 42, 43]. Here, we demonstrated that 9V/null mice had progressive accumulation of substrates in the brain, predisposing to brain pathology including αSyn and p-Tau accumulation. The 9V/null mice developed cognitive deficits toward both non-aversive stimuli (repeated open field) and stress (fear test). Interestingly 9V/null mice exhibited abnormal innate, anxiety-like behavior with a biphasic phenotypic expression for early and late onsets (6 months vs. 18 months of age). Abnormal motor function and hepatosplenomegaly were evident in maturing 9V/null mice, both with more significant and more consistent penetration in females than in male mice. This chronological evaluation of 9V/null mice identifies CNS functional abnormalities that are progressive with substrate accumulation and brain pathology in an age-dependent and/or gender-dependent manor. Thus, 9V/null mice present the phenotype as a chronic nGD mouse model.

*GBA 1* mutations in homo- and heterozygosity have been associated with high risk for PD development in humans [12, 14, 20]. Neurological evaluation of patients with “non-pathogenic” GD type 1 and carriers has revealed that they may develop cognitive impairment at advanced age that is associated with established PD [18, 44, 45]. Memory impairments are also demonstrated in *Gba1* mutant mice [30, 32]. Consistent with these reports, short-term spatial memory deficit was demonstrated by 9V/null mice in the repeated open-field test, supporting the role of *GBA1* mutation in the development of PD. Our longitudinal study determined the onset of this memory deficit as 9 to 12 months of age, signifying a slow and progressive nature of the neurological signs that have been described in human and mice with GD-associated PD [18, 32, 44, 45].

Characterizing substrate levels is essential for understanding disease pathogenesis and evaluation of therapies. There are only few reports on the substrate levels in brains from patients with GD type 1 [46, 47]. Due to the limited access to human samples, it is very difficult to conduct a longitudinal study on quantitation of substrate levels in human brains. Such a study has to be conducted in the viable *Gba1* mutant mice, e.g. 9V/null mice that present with the mild chronic GD phenotype [29]. The longitudinal analysis of GC/GS has been studied in visceral tissues in 9V/null mice until 1 year of age and shows progressively elevated substrates with age [42]. In this study, excess GC accumulation progressed with age and increased substantially in the 2^nd^ year of life in the brain of 9V/null mice. GS, which is absent or at very low levels in WT brains throughout life, was detectable in 9V/null brains as early as 3 months of age, and escalated progressively with the aging process. Thus, GS levels would be a more sensitive marker associated with an earlier onset than GC quantification. This finding likely suggests that progressive accumulation of the substrates is expected in the brain of chronic GD patients that have a normal life expectancy.

Accumulation of the substrates, GC and its deacylated form GS, is toxic to neural cells and is thought to play a major role in neurodegeneration [11, 46, 48, 49]. GS can mobilize calcium [49] and the accumulation of GC causes excess calcium efflux from the endoplasmic reticulum [50]. Elevated cellular calcium levels have also been observed in iPSC-derived neurons from GD type 1 patients, indicating that impaired calcium homeostasis may play a role in neurodegeneration in nGD [51]. Excess substrates also have the potential to harm mitochondrial function which has been shown in nGD cells and mouse models. In a human dopaminergic cell line, inhibition of GCase by the covalent inhibitor, conduritol B epoxide, leads to substrate accumulation and defective mitochondrial function [23]. Impaired respiratory chain and mitochondrial membrane potential are also detected in primary neurons and astrocytes derived from an acute mouse model of nGD [52]. In nGD mouse brains that have accumulated GC and GS, the isolated brain mitochondria show decreased ATP production and oxygen consumption that are critical for neuron functions [25]. In addition, excess GC and GS also impair lysosomes and autophagy resulting in macromolecule aggregation e.g. APP and αSyn [25, 53]. These defective cellular functions mediated by substrate accumulation could underlie the observed behavioral impairment and motor function deficits.

An abnormally elevated αSyn level, which is the biochemical signal of GD mutation for susceptibility to PD [11, 54–56], was detected in 9V/null brain. Increased αSyn may inhibit GCase activity by interaction with the enzyme [57]. It has been suggested that there is a positive feedback between toxic αSyn monomer formation mediated by excess GC in lysosomes and further GCase reduction by inhibition via αSyn accumulation [11]. The increased αSyn together with substrate accumulation has been reported in rapid neurological progressive GD models and another chronic GD model (9V/9V) [11, 15, 25, 32, 58]. Similar to these models, the pathological accumulation of αSyn in 9V/null is predominantly in the hippocampal and cortical regions that are associated with memory. In the same regions, increased p-Tau was also detected in 9V/null mice. Pathological aggregation of Tau protein is implicated in other neurodegenerative diseases and in the brains of GD type 3 patients [4, 59]. Tau has a physiological interaction with αSyn indicating its role in synucleinopathies [60]. Several studies have shown a synergistic relationship between tau and αSyn that promotes their mutual aggregation, phosphorylation and accumulation and accelerates cognitive decline in neurodegenerative disorders, such as Alzheimer’s disease or dementia with Lewy body disease [61–63]. In this longitudinal study, αSyn was above normal levels starting at 6 months of age in 9V/null brain, while elevated p-Tau signals emerged at 12 months. In correlation, 9V/null mice showed impairment of conditioned learning at 12 months of age in a conditioned fear task, a classic approach to evaluate emotional cognitive memories to learn the association of a neutral conditional stimulus (tone) with an aversive stimulus (electric shock) [64, 65]. Regardless of gender, 9V/null mice exhibited significantly less defensive response (freezing) to the cued stimuli. Several brain regions are associated with emotion, including the amygdala, amygdalo-hippocampal area, peri-/entorhinal cortex, and hypothalamus [66]. Elevated αSyn and/or p-Tau signals have been observed in these relevant regions [4, 55]. In addition, it has been reported that Tau promotes or enables the development of learning and memory deficits [67]. Thus, Tau pathology is likely to be a more direct indication of cognitive deficits in nGD. However, αSyn is another factor, in addition to excess substrates, that could contribute to the neuronal deficits in 9V/null mice.

The progression of visceral symptoms with age often affects stress levels in animals with LSDs, and changes in anxiety can affect learning and memory tasks [36]. The marble-burying task allows assessment of non-associative, impulsive/compulsive or anxiety-related behavior. The 9V/null mice were hypersensitive (especially female) to marbles and began digging activities much more quickly than normal controls at relatively young age (< 9 months). However, this sensitivity may not be due to abnormal obsession/compulsion, but rather to general anxiety. This is because no difference was detected in the numbers of marbles buried, which seem to be more consistently related with repetitive obsessive/compulsive behavior than other parameters [68, 69]. With disease progression, 9V/null mice became hyposensitive to marbles at 12 months of age or older in both genders. This slower response to marbles may be related to progressive visceral problems (hepatosplenomegaly) in older 9V/null mice. However, increased anxiety was only observed in female 9V/null mice. This disparity between sexes in anxiety levels may be related to the earlier onset of hepatosplenomegaly we observed in female 9V/null mice. Thus increased anxiety levels in female 9V/null mice may have contributed in part to their decreased spatial learning and memory deficits.

Abnormal sensorimotor function demonstrated by abnormal gait is a common neurological sign in acute nGD in human and mouse models [8, 70, 71]. It has been reported that abnormal sensorimotor function contributes to myoclonus which has been observed in patients with nGD [72]. In gait analysis, abnormally wider paw-bases detected in 9V/null mice were more consistent in females with earlier onset (3 or 9 months of age for front or hind-paws, respectively) than in males (12 or 18 month of age). This is different from male-prone gait symptoms reported in PD patients or drug-induced PD mouse model that are likely associated with gonadal steroids rather than loss of dopaminergic neurons [73, 74]. Moreover, increased stride length, which is normally correlated with heavier body weight, was found in female 9V/null mice than all other groups at either relatively younger ages (3 and 6 months) or older than 18 months when their body weights were less than male groups and similar/less than age-matched normal female mice. The transient disappearance of longer stride length at 9 and 12 months of age may be due to significantly less body weight in female 9V/null mice emerging at 9 months of age that counteract with longer strides. The motor behavior is often expressed through the interactions of several physiologic systems (neurologic, cardiovascular and musculoskeletal) and affected by emotional and/or physical stress. For example, limb symptoms of rapid-onset dystonia Parkinsonism are often triggered by physical and/or emotional stress [75]. The longer stride length seems to be associated with increased anxiety in female 9V/null mice. For evaluating motor function deficits, the 9V/null female would be the preferred gender for sensitive detection of gait abnormalities. Gait/footprint analysis has been used to assess sensorimotor function in Thy1-αSyn mice, a PD mouse model [76]. Notably, in this study, the abnormal sensorimotor function demonstrated by gait analysis is correlated with αSyn aggregation detected in the sensorimotor region of the brain from 9V/null mice, although progressive skeletal disease or myoclonus may contribute partially to the gait abnormities in this chronic nGD mouse model. This abnormal CNS function is also in line with the observation that hind-limb paralysis was one of the unique characteristics in a severe/acute nGD mouse model which only has a life-span of 48 days without much visceral complications [71].

Gender differences in behavioral performance and/or neurotoxicity have been reported in some lysosomal storage disease (LSD) models such as MPS type I or IIIA mice [36, 77, 78]. Studies on hormone therapeutic effects have shown that higher estrogen levels could result in significantly improved neurological symptoms and greater longevity in Saposin A^-/-^-deficient mice, which is a model for late-onset globoid cell leukodystrophy [79]. The administration of estradiol was reported to inhibit brain macrophages [80], while macrophage activation was implicated in the CNS pathology in nGD mice [81, 82], as well as several other LSD models including MPS IIIB [83], metachromatic leukodystrophy [84] and Niemann-Pick disease type C [85]. This may explain, in part, the higher vulnerability found in male 9V/null mice that exhibited an abnormal defensive freezing toward contextual fear stimuli, and a much deeper reduction in response toward cured fear stimuli than female counterparts. These observations are in agreement with other studies showing that female animals were insensitive to stress paradigms known to affect the males [86–88]. In contrast, increased anxiety presented by marble burying activities were observed only in female 9V/null mice. Disparate effects in response to chronic stress have been reported between male and female animals [86, 89]. In addition to hormone-receptor interaction, gender, as a CNS impairment variable, also involves both genomic and non-genomic factors [90–92].

The disease-specific treatment approaches for GD patients include ERT and SRT [9, 10, 93]. However, the recombinant enzyme is unable to pass the blood-brain barrier in therapeutically effective amounts, and the SRTs that inhibit glucosylceramide synthase have not shown effectiveness in moderating the neurological phenotype [9,94–96]. Several CNS-targeted approaches have recently emerged demonstrating CNS efficacy in preclinical mouse models with LSDs [96–99], and hold a promise for treatment of nGD. This study documents a temporal, systematic evaluation on the phenotypic expression of behavioral dysfunctions and brain pathogenic abnormalities in 9V/null mice, validating it as a chronic nGD mouse model. The data characterize chronological behavioral and gait profiles in 9V/null mice from adolescence through maturity to advanced age, and identify the onsets of functional deficits and their interaction with genders. These biochemical, pathological, behavioral and gait assessments will provide guidelines for experimental designs to evaluate potential CNS therapies for the treatment of nGD.

## Supporting Information

S1 Fig Changes of body weight in male and female 9V/null and WT mice during development. N = 6–16 mice per age group. *, p<0.05; **, p<0.01 by Student’s t-test.
(PDF)

S2 Fig Distribution of αSyn and p-Tau in the brain of WT and 9V/null mice. A: αSyn (violet) signals in 9V/null hippocampus, thalamus and cerebellum. B: Distribution of αSyn aggregates in neurons and astrocytes. Representative views are shown (left panel) for αSyn aggregates (green, arrows, >2 μm in size) in neurons (Map2^+^, red) or in astrocytes (GFAP^+^, red). Semi-quantitation analysis was conducted by counting cells from 10–12 fields per brain section that include 4 regions (cortex, mid-brain, brain stem and thalamus). The average percentages of αSyn-containing neurons (14.4%) or astrocytes (4.3%) in 9V/null brain were significantly greater than those in WT brain (7.9% and 2.9%, respectively). P values are from Student’s t-test. C: p-Tau signals (violet) detected in 9V/null cortex and hippocampus. The brain sections were from 12-months old mice. DAPI (blue) stains nuclei.
(TIFF)

S3 Fig Brain pathology by immunohistochemistry analysis with CD68 staining. Brain was harvested from WT and 9V/null mice at 20 months of age after trans-cardiac perfusion. Slides from frozen-sections were used for immunohistochemistry staining of CD68. A: Representative pictures for selected regions, showing brown spots as CD68-positive signals. B: Semi-quantitative analysis in six brain regions using Image J software. For each region, data are derived from eight pictures (20x) randomly taken from brains of two mice per group. CBR, cerebellum; DCN, deep cerebellar nuclei. There is no significant difference between WT and 9V/null. The scale bar is 200 μm.
(PDF)

S1 Table Parameters from complete blood counts in 9V/null and strain-matched WT mice. (PDF)
