# Supplementary material for: Progression of Behavioral and CNS Deficits in a Viable Murine Model of Chronic Neuronopathic Gaucher Disease
Source: PLoS One. 2016 Sep 6;11(9):e0162367. doi: 10.1371/journal.pone.0162367 (PMC5012639; doi:10.1371/journal.pone.0162367)
Supplement: S1 Fig — N = 6–16 mice per age group. *, p<0.05; **, p<0.01 by Student’s t-test. (PDF) [file pone.0162367.s001.pdf]

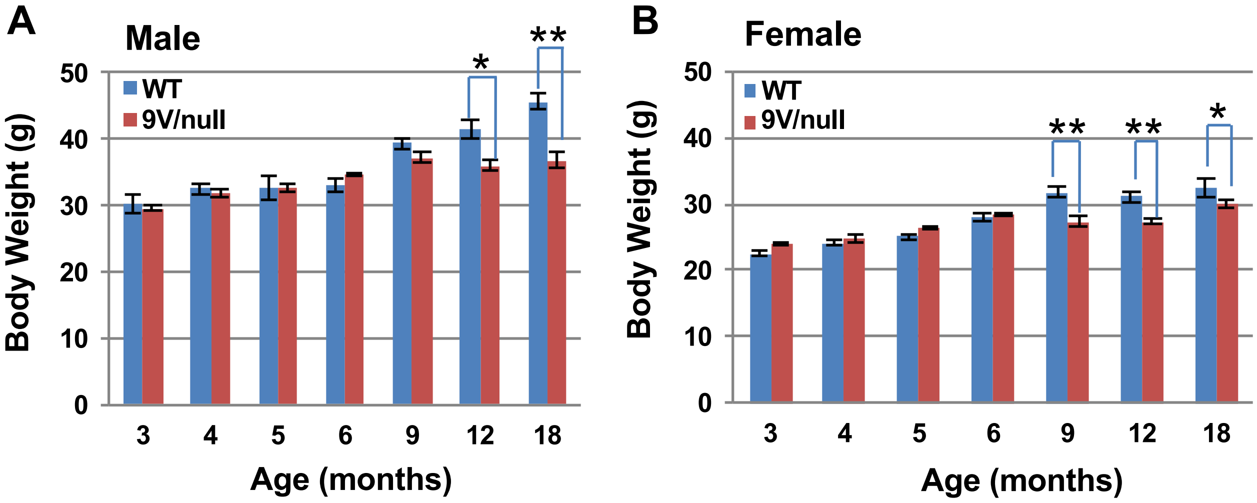

S1 Fig. Changes of body weight in male and female 9V/null and WT mice during development. N = 6-16 mice per age group. *, p<0.05; **, p<0.01 by Student's t-test.
