# Supplementary figures and images for: Progression of Behavioral and CNS Deficits in a Viable Murine Model of Chronic Neuronopathic Gaucher Disease
Source: PLoS One. 2016 Sep 6;11(9):e0162367. doi: 10.1371/journal.pone.0162367 (PMC5012639; doi:10.1371/journal.pone.0162367)

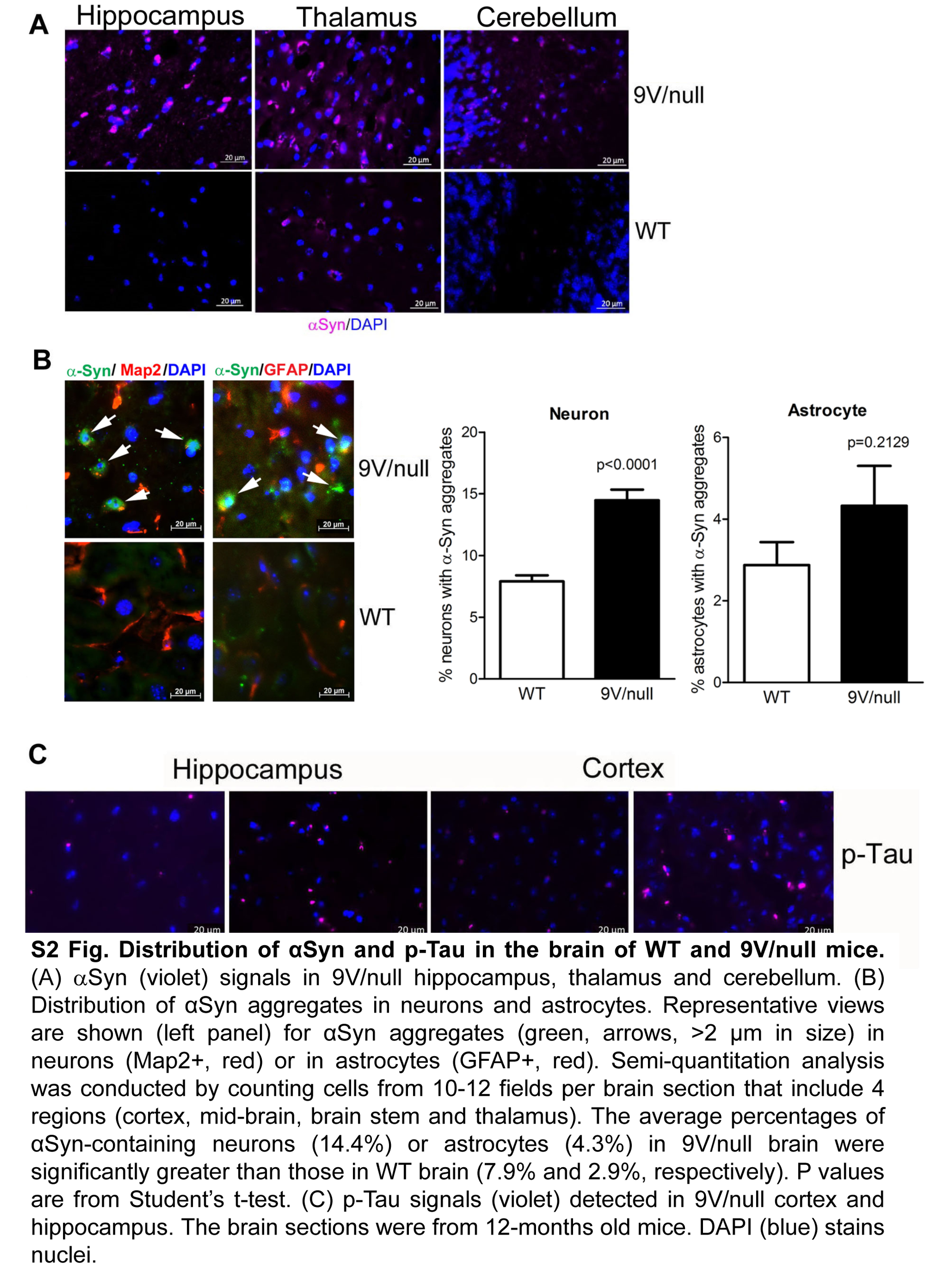

Supplement: S2 Fig — A: αSyn (violet) signals in 9V/null hippocampus, thalamus and cerebellum. B: Distribution of αSyn aggregates in neurons and astrocytes. Representative views are shown (left panel) for αSyn aggregates (green, arrows, >2 μm in size) in neurons (Map2+, red) or in astrocytes (GFAP+, red). Semi-quantitation analysis was conducted by counting cells from 10–12 fields per brain section that include 4 regions (cortex, mid-brain, brain stem and thalamus). The average percentages of αSyn-containing neurons (14.4%) or astrocytes (4.3%) in 9V/null brain were significantly greater than those in WT brain (7.9% and 2.9%, respectively). P values are from Student’s t-test. C: p-Tau signals (violet) detected in 9V/null cortex and hippocampus. The brain sections were from 12-months old mice. DAPI (blue) stains nuclei. (TIFF) [file pone.0162367.s002.tiff]
