# Supplementary material for: Progression of Behavioral and CNS Deficits in a Viable Murine Model of Chronic Neuronopathic Gaucher Disease
Source: PLoS One. 2016 Sep 6;11(9):e0162367. doi: 10.1371/journal.pone.0162367 (PMC5012639; doi:10.1371/journal.pone.0162367)
Supplement: S3 Fig — Brain was harvested from WT and 9V/null mice at 20 months of age after trans-cardiac perfusion. Slides from frozen-sections were used for immunohistochemistry staining of CD68. A: Representative pictures for selected regions, showing brown spots as CD68-positive signals. B: Semi-quantitative analysis in six brain regions using Image J software. For each region, data are derived from eight pictures (20x) randomly taken from brains of two mice per group. CBR, cerebellum; DCN, deep cerebellar nuclei. There is no significant difference between WT and 9V/null. The scale bar is 200 μm. (PDF) [file pone.0162367.s003.pdf]

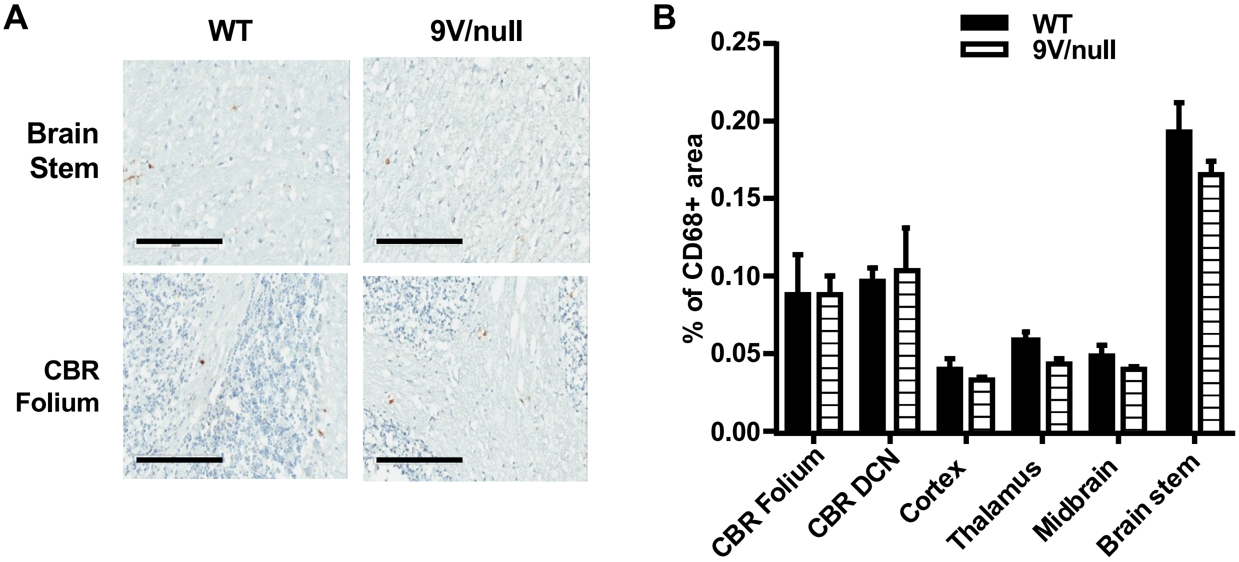

S3 Fig. Brain pathology by immunohistochemistry analysis with CD68 staining. Brain was harvested from WT and 9V/null mice at 20 months of age after trans-cardiac perfusion. Slides from frozen-sections were used for immunohistochemistry staining of CD68. A: Representative pictures for selected regions, showing brown spots as CD68-positive signals. B: Semi-quantitative analysis in six brain regions using Image J software. For each region, data are derived from eight pictures (20x) randomly taken from brains of two mice per group. CBR, cerebellum; DCN, deep cerebellar nuclei. There is no significant difference between WT and 9V/null. The scale bar is 200 μm.
