# Supplementary material for: Progression of Behavioral and CNS Deficits in a Viable Murine Model of Chronic Neuronopathic Gaucher Disease
Source: PLoS One. 2016 Sep 6;11(9):e0162367. doi: 10.1371/journal.pone.0162367 (PMC5012639; doi:10.1371/journal.pone.0162367)
Supplement: S1 Table — (PDF) [file pone.0162367.s004.pdf]

S1 Table. Parameters from complete blood counts in 9V/null and strain-matched WT mice*.

		9-months		15-months		24-months	
		Avg ±SD	n	Avg ±SD	n	Avg ± SD	n
**WBC (k/μL)**	WT	2.02 ± 1.2	25	2.07 ± 1.5	24	1.89 ± 0.72	5
	9V/null	1.60 ± 1.1	19	2.21 ± 0.9	27	2.51 ± 0.97	14
**RBC (M/μl)**	WT	9.70 ± 2.3	25	10.6 ± 1.4	24	10.8 ± 0.37	5
	9V/null	9.80 ± 2.1	19	10.1 ± 1.8	27	10.5 ± 2.4	14
**Platelet (K/μL)**	WT	799 ± 227	25	727 ± 256	24	968 ± 339	5
	9V/null	890 ± 290	19	971 ± 249	27	1091 ± 457	14

* Total blood counts were performed using DREW HEMAVET with blood samples from trans-cardiac collection.
